# Factors Associated With Being Overweight or Obese in Suriname

**DOI:** 10.3389/ijph.2021.1604101

**Published:** 2021-06-11

**Authors:** Jeetendra Khadan, Nekeisha Spencer, Eric Strobl, Theophiline Bose-Duker

**Affiliations:** ^1^ Inter-American Development Bank, Washington, DC, United States; ^2^ Department of Economics, University of the West Indies, Kingston, Jamaica; ^3^ Department of Economics, University of Bern, Bern, Switzerland; ^4^ Department of Economics, University of Birmingham, Birmingham, United Kingdom

**Keywords:** public health, BMI, overweight, Suriname, policy

## Abstract

**Objective:** To identify the socio-demographic risk factors that are associated with adult Body Mass Index.

**Methods:** We apply probit and ordinal probit models to a sample of 3,803 adults aged 20 and above from the 2016/17 round of the Suriname Survey of Living Conditions.

**Results:** Women, the elderly, and couples who are either married and/or living together are more likely to be obese or overweight. This is also true for individuals who have chronic illnesses. We also find that individuals who engage in a sport or in other forms of exercise, even if modest, have lower odds of being overweight or obese. Interestingly, our findings indicate that individuals who benefit from government social safety net programs are less likely to be associated with being overweight or obese.

**Conclusion:** Obesity could become a serious public health issue if not addressed appropriately. Policymakers should promptly develop a national strategy to help health care systems cope with the outcomes of obesity and to tackle the risk factors that have the greatest impacts on individual Body Mass Index.

## Introduction

Being overweight is one of the leading risk factors of premature death. It is estimated to cause an average of 3·9% of years of life and 3·8% of disability-adjusted life-years (DALYs) worldwide [1]. Alarmingly, its prevalence appears to be rapidly rising [2]. While traditionally the disease was viewed as a developed world’s problem as it was strongly associated with income, with rising incomes, changing eating habits, and reduced physical activity in low- and middle-income countries, it is now a common phenomenon worldwide [3, 4]. As a matter of fact, according to the latest figures available from the World Health Organization, at least 39% of adults are currently overweight worldwide, and about 26% in low-income and lower-middle income countries.

One region that appears to be particularly aﬀected in the developing world is the Caribbean. For instance, within the Caribbean Community and Common Market (CARICOM), more than 60% of adults are overweight and this number is as high as 80% in some countries like Barbados [5]. Suriname has been noted as a high-risk obesity country experiencing rapid growth in adult obesity over the two-decade period, 1995–2015. Further, it has been cited as having very poor chances of meeting the 2025 United Nations adult obesity targets where obesity predictions for the country by this time would reach at approximately 40% for women and 26% for men compared to almost 10 percentage points lower in 2010 [6].

Signs of the consequences of the high incidence of the obesity problem are already apparent in the prevalence of, for instance, diabetes - which ranges between 10 and 15% as compared to the 9.0% and 9.5% averages for low-income and lower-middle income countries as a whole - and has been a major contributor to pre-mature deaths in the region [7]. Similarly, others find that being overweight can explain 26% of the variance in blood pressure in females and 13% in males in the islands of Jamaica, St. Lucia, and Barbados [8]. Given the status quo and future obesity outlook for Suriname, diseases associated with being overweight will form part of the burden on the health system. As a result, it is important to understand the factors driving obesity in Suriname to develop appropriate policies to reduce growth in obesity.

Despite the expanding literature on the determinants of obesity across the globe [9–13], the apparent widespread weight problem in the Caribbean and the number of studies regarding what might be driving its prevalence is rather limited. For instance, misperceptions, inactivity and maternal factors may drive obesity among Barbadian adolescents [14], while marital status and cigarette smoking factors predicted the Body Mass Index (BMI) for both male and female urban Jamaican African origin adults [15]. In the case of Jamaica, fast-food and sweetened beverage consumption were associated with overweight and high waist circumference in adolescents [16]. For Bahamas, education was the strongest (negative) predictor of obesity prevalence [17]. In a study of school aged children in Haiti, found that always purchasing food at school, the mother’s BMI and household ownership of a bicycle were significant predictors of being overweight [18].

In this study we further add to the scarce literature on the factors associated with being in the Caribbean by investigating this for the case of Surinamese adults. To our knowledge, there is only one other study that has examined the determinants of any weight problem in Suriname. More specifically, data from the Healthy Life in Suriname (HELISUR) study shows that the obesity prevalence ratio was significantly lower in participants meeting the WHO physical activity recommendations, in particular those regarding commuting and leisure time domains [19]. The authors discovered that active minutes/week and total volume of activity were negatively associated with obesity for all adults in the African-Surinamese members but not in the Asian-Surinamese population. In this paper, we use the 2016/2017 Suriname Survey of Living Conditions to examine the role of socio-demographic factors on BMI among persons living in SurnameAs it relates to the region, our study adds to the literature by highlighting that even modest forms of exercise can be significant in lowering one’s weight. A more striking result, however, is that the fact that beneficiaries of public safety net programs are less likely to be overweight. Our results are key to developing a multidimensional policy to successfully address the problem of obesity in Suriname.

## Methods

### Data

This study uses the 2016/17 round of the nationally representative Suriname Survey of Living Conditions (SSLC). The goal of the survey is to collect data on various socio-demographic variables to assist in poverty analysis, to support the policy-making process and to develop objective baseline indicators for the design of IDB projects. Data was collected over a period of 1 year to account for seasonality using a two-stage sampling process. About 2,100 households from all 10 districts of the country were surveyed using extensive questionnaires on education, health, child anthropometrics, access to social services, fertility, employment, housing, emigration, income, consumption and financial inclusion. Our sample consists of 3,803 adults aged 20 and above.

For our purposes, the SSLC 2016/17 allows us to account for various demographic, social and economic factors including rurality, physical activity, socioeconomic status, labor market outcomes, income, level of education and assets ownership including land, television sets and computers. BMI is also easily calculated for everyone as data on height (centimeters) and weight (kilograms) is collected for each individual surveyed.

We apply the standard WHO cut-oﬀ values for BMI for the Western World (Latin America and the Caribbean are considered a part of the Americas). Individuals with BMI values below 18.5 are considered underweight, those with values between 18.5 and 24.9 are considered to be of normal weight, those with values between 25 and 29.9 are considered overweight and those with BMI values of 30.0 and above are considered to be those with obesity. On the other hand, data on individuals aged 20 to 65 were used to construct optimal ethnic and sex-specific cut-oﬀ values for obese adults in Suriname [20]. These BMI cut-oﬀ values are derived specifically for the prediction of hypertension and cardio-metabolic risk. The results from the study show that these cut-oﬀ values vary significantly by sex and ethnic groups. For instance, for hypertension, the optimal cut-oﬀ values for adult male obesity are 28.4, 24.8, 25.2, 25.5, 25.0 and 25.9 for Amerindian, Creole, East Indian, Javanese, Maroon and Mixed men respectively. For cardio-metabolic risk, they find optimal cut-oﬀ values of 26.5, 25.4, 25.0, 25.5, 25.5 and 25.1 for Amerindian, Creole, East Indian, Javanese, Maroon and Mixed men respectively. These values are generally higher for women belonging to the respective ethnic groups. We compare our results to estimates using these cut-oﬀ values and find that our results do not change substantially.

The variables used in our study can be put into three categories: individual characteristics, economic, and health & physical activity.

#### Individual Characteristics

For this group, the SSLC 2016/17 allows us to include the gender of the individual, marital status, age and one’s level of education. We also include the ethnic group the individual belongs to as diﬀerent ethnic groups may have diﬀerent diets and eating habits which could have an impact on BMI.

#### Economic Variables

These variables capture the level of economic activity that each individual and/or household is involved in. To account for the labor market status of everyone, we include employment status and total individual income. We also include a variable that captures public transfers from the government for individuals who benefit from government safety net programs. The number of young children present in each household is also included as having young children could be a barrier to labor force participation especially for women due to domestic caring responsibilities [21]. To account for asset ownership and wealth, we include the ownership of arable or homestead land by each household and the number of television sets and desktop computers owned by each household. Finally, we attempt to capture rurality by including variables that indicate whether an individual lives in the capital city, Paramaribo and its environs or in the interior regions.

#### Health and Physical Activity

We capture the health status of each individual with a variable that indicates whether he/she has at least one of these chronic conditions: diabetes, high blood pressure, asthma, arthritis and cancer. We also account for the activity levels of individuals with a variable that indicates whether an individual engages in physical activities such as regular walking, jogging, horse riding, exercising in a gym or any other sporting activity.

### Empirical Model

To investigate the relationship between the various socio-economic variables and being overweight, we run 2 probit models: a probit regression and an ordered probit regression. We use both models because we investigate 2 dependent variables. The dependent variable for the probit model is a dummy variable which is equal to 1 if the individual’s BMI is greater than or equal to 25, that is, if the individual is overweight. For the ordered probit model, the dependent variable is a naturally ordered discreet variable which is equal to 1 if the individual is underweight, 2 if the individual is of normal weight and 3 if the individual is overweight. The ordered probit model is a generalization of the widely used probit model where the dependent variable has more than 2 outcomes [22, 23].

Both models are specified as follows:

$$Y_{ij}=F\left(\alpha +\beta_{1}C_{ij}+\beta_{2}H_{ij}+\beta_{3}A_{ij}\right)$$ (1)

where *F* is the standard normal cumulative distribution function and the subscripts *i* and *j* denote individual and household respectively. *Y*_*ij*_ represents the dependent variable which is some representation of the BMI category the individual falls within, *α* represents the constant, *C*_*ij*_ is a vector of individual characteristics, *H*_*ij*_ is a vector of household characteristics, and *A*_*ij*_ is a vector of community characteristics. All standard errors are clustered at the household level to account for within-household correlation that may exist between individuals living in the same household.

We use the same set of independent variables for both models to facilitate easy comparison. The vector of individual characteristics (*C*_*ij*_) includes a dummy variable which is equal to 1 if the individual is female, a dummy variable equal to 1 if the individual is married or living together with his/her partner, age, age squared, one’s level of education, a dummy variable equal to 1 if the individual is employed, the log of total individual income, a dummy variable equal to 1 if the individual benefits from at least 1 government social safety net program and a dummy variable equal to 1 if the individual has one of the following chronic conditions: diabetes, high blood pressure, asthma, arthritis and cancer. We also account for the activity levels of individuals by including a dummy variable that is equal to 1 if an individual engages in at least one of the following physical activities: exercises in a gym at least once a week, engages in any sporting or physical activity at least once a week, goes jogging at least once a week, takes regular walks at least three times a week or rides with a riding club at least once a week. Finally, we capture ethnicity using dummy variables which indicate whether an individual is Asian (includes East Indian, Javanese and Chinese), African (includes Creole and Maroon), Indigenous, White, Mixed with all other ethnic groups grouped together (Other) as the reference category.

The vector of household characteristics (*H*_*ij*_) includes the number of television sets and desktop computers owned by the household, the number of children aged 0 to 5 within the household and a dummy variable indicating that the household owns arable or homestead land. Finally, the vector of community characteristics (*A*_*ij*_) is made up of a group of dummy variables that indicate whether an individual lives in the capital Paramaribo and its environs or in Sipaliwini and its environs which is mainly forest. The other districts are grouped together and act as the reference category.

## Results

### Summary Statistics

Table 1 presents sample statistics according to each BMI category. 4% of the sample is underweight (BMI is less than 18.5), 38% of the sample falls within the normal weight category (BMI between 18.5 and 25), 36% of the sample is overweight (BMI between 25 and 30) while 22% of the sample are obese (BMI is greater than 30). These statistics are worrying since more than half of the sample is either overweight or obese. There are a few interesting patterns worth mentioning from Table 1 and the diagrams. First, women are more likely than men to be on the extremes of the BMI scale, that is, they are more likely to be underweight or obese while men are more likely to be of normal weight or overweight. Surprisingly, the percentage of our sample who are overweight is slightly higher for younger adults (aged 20–59) than for older individuals (aged 60 and above). From Table 1, we also see that individuals who are married or living together are more likely to be overweight. The same is true for individuals who have a chronic illness. On average, more than 60% of the individuals who fall within the underweight category are of Asian origin. Finally, the category with the largest proportion of individuals who benefit from government social safety net programs is the underweight category.

**TABLE 1:** Summary statistics for sample by body mass index categories, Suriname, 2016–2017.

	Underweight (BMI<18.5)	Normal weight (18.5 ≤ BMI<25)	Overweight (25 ≤ BMI<30)	Obese (BMI ≥ 30)	Total
Female	0.55	0.47	0.47	0.62	0.51
	(0.50)	(0.50)	(0.50)	(0.48)	(0.50)
Married/living	0.39	0.56	0.64	0.67	0.61
Together	(0.49)	(0.50)	(0.48)	(0.47)	(0.49)
Age	43.3	46.0	46.4	46.9	46.2
	(19.66)	(16.55)	(14.92)	(14.30)	(15.65)
Has completed	0.43	0.44	0.46	0.43	0.44
VOJ^a^	(0.50)	(0.50)	(0.50)	(0.49)	(0.50)
Lives in	0.46	0.38	0.40	0.36	0.39
Paramaribo	(0.50)	(0.49)	(0.49)	(0.48)	(0.49)
Asian	0.62	0.55	0.52	0.48	0.53
	(0.49)	(0.50)	(0.50)	(0.50)	(0.50)
African	0.20	0.28	0.30	0.33	0.30
	(0.40)	(0.45)	(0.46)	(0.47)	(0.46)
Indigenous	0.04	0.02	0.02	0.02	0.02
	(0.20)	(0.16)	(0.16)	(0.15)	(0.15)
Mixed	0.13	0.14	0.14	0.15	0.14
	(0.34)	(0.35)	(0.34)	(0.36)	(0.35)
White	0.000	0.004	0.003	0.000	0.003
	(0.00)	(0.06)	(0.05)	(0.00)	(0.05)
Individual is	0.48	0.62	0.66	0.60	0.63
Employed	(0.50)	(0.49)	(0.47)	(0.49)	(0.48)
Individual is	0.50	0.53	0.48	0.49	0.50
Active	(0.50)	(0.50)	(0.50)	(0.50)	(0.50)
Individual receives	0.11	0.06	0.05	0.07	0.06
gvt assistance	(0.32)	(0.24)	(0.21)	(0.25)	(0.23)
Individual has a	0.20	0.27	0.32	0.46	0.33
Chronic disease	(0.40)	(0.45)	(0.47)	(0.50)	(0.47)
Number of tv sets and	1.09	1.25	1.27	1.24	1.25
Desktops in hhs	(0.69)	(0.73)	(0.73)	(0.70)	(0.72)
Sample size	152	1,447	1,365	839	3,803

Standard deviations are in parentheses.

a VOJ refers to general junior secondary education.

Table 2 presents the results from both the probit and ordered probit estimations in columns 2 and 4 respectively. The results are quite consistent across both models especially regarding significance and the signs of the coeﬃcients. The actual values of the coeﬃcients are also reasonably similar. Column 3 presents the marginal eﬀects of each of the variables from the probit model. The marginal eﬀects from the ordered probit model are similar to those of the probit model. Hence, for a cleaner presentation, we present the marginal eﬀects of only the probit model. It is worth mentioning that our results only show associations between each variable (while controlling for as many factors as possible) and the probability of being overweight. They do not indicate a causal relationship.

**TABLE 2:** Factors associated with being overweight/obese - Probit models, Suriname, 2016–2017.

	Probit model		Ordered probit
	Estimates	Margins	Estimates
Female	0.149^∗∗∗^	0.0560^∗∗∗^	0.127^∗∗∗^
	(0.0445)	(0.0166)	(0.0422)
Married/living together	0.227^∗∗∗^	0.0849^∗∗∗^	0.241^∗∗∗^
	(0.0474)	(0.0176)	(0.0457)
Age	0.0485^∗∗∗^	0.0182^∗∗∗^	0.0527^∗∗∗^
	(0.00813)	(0.00301)	(0.00781)
Age squared	-0.000498^∗∗∗^	-0.000187^∗∗∗^	-0.000531^∗∗∗^
	(0.0000824)	(0.0000305)	(0.0000791)
Lives in capital	-0.0136	-0.00512	-0.0434
	(0.0493)	(0.0185)	(0.0483)
Lives in interior	0.106	0.0396	0.0973
	(0.128)	(0.0478)	(0.131)
Asian	-0.364^∗^	-0.137^∗^	-0.273
	(0.192)	(0.0718)	(0.220)
African	-0.103	-0.0386	0.0106
	(0.194)	(0.0726)	(0.221)
Indigenous	-0.0851	-0.0319	-0.0755
	(0.238)	(0.0892)	(0.265)
Mixed	-0.188	-0.0706	-0.0825
	(0.197)	(0.0737)	(0.223)
White	-0.636	-0.238	-0.414
	(0.484)	(0.181)	(0.412)
Employed	0.0228	0.00856	0.0535
	(0.0713)	(0.0267)	(0.0693)
Log of total income	0.00481^∗^	0.00180^∗^	0.00476^∗^
	(0.00292)	(0.00109)	(0.00285)
Active individual	-0.103^∗∗^	-0.0384^∗∗^	-0.0772^∗^
	(0.0457)	(0.0171)	(0.0452)
No of tv sets in household	0.0137	0.00514	0.0400
	(0.0328)	(0.0123)	(0.0317)
Level of education	-0.00411	-0.00154	-0.00908
	(0.0222)	(0.00833)	(0.0216)
No of 0–5years olds in household	0.0560	0.0210	0.0523
	(0.0377)	(0.0141)	(0.0362)
Household owns agric land	-0.172	-0.0644	-0.119
	(0.124)	(0.0464)	(0.108)
Receives gvt assistance	-0.182^∗∗^	-0.0681^∗∗^	-0.220^∗∗^
	(0.0907)	(0.0339)	(0.0869)
Has a chronic condition	0.325^∗∗∗^	0.122^∗∗∗^	0.321^∗∗∗^
	(0.0502)	(0.0185)	(0.0478)
*N*	3,803	3,803	3,803

Standard errors in parentheses.

*p < 0.10, **p < 0.05, ***p < 0.01.

From Table 2, the coeﬃcient on female is positive and significant at 1% for both models. This means that women in Suriname have a higher risk of becoming overweight. The marginal eﬀect of female in the probit model (column 3) shows that compared to men, the predicted probability of being overweight for a Surinamese woman increases by 5.6%. Our findings also indicate that the prevalence of obesity and overweight is much lower amongst single individuals. Single individuals include individuals who have never married, are widowed, separated or divorced. This result is highly significant in both regression models. The marginal eﬀect from the probit model shows that the predicted probability of being overweight increases by 8.5% if an individual is married or shares a residence with his or her partner.

Similar to some authors, the relationship between age and individual BMI levels appears to be concave with a maximum turning point of 48.69 [24–26]. This implies that the chances that an individual will become overweight increases as the person gets older up till age 48. After age 48, the relationship between BMI and age becomes negative. As expected, our findings also indicate that chronic conditions such as diabetes, high blood pressure and cancer are strongly associated with being overweight. The predicted probability of being overweight increases by 12.2% for adults who have at least one of these conditions. We also find that active individuals are generally less likely to be overweight. In particular, the predicted probability of being overweight for individuals who engage in sporting activities at least once a week, workout in a gym at least once a week or take a walk at least three times a week falls by about 3.8%. In addition, we show that individuals who benefit from government social safety net programs are less likely to be overweight. The predicted probability of being overweight for these individuals decreases by 6.8%. According to the probit model, it also appears that the predicted probability of being overweight falls by 13.7% for individuals of Asian origin.

We also carry out our regressions separately for men, women, the young (aged 20–59 years old) and the old (aged 60 and above). The results from these regressions are presented in Table 3 (male and female) and 4 (young and old). There are a few patterns worth mentioning from these 4 tables. First, from Table 3 women (unlike men) with higher income levels have a larger propensity to be overweight. Second, Table 4 shows that the predicted probability of being overweight increases by 3.11% for young adults who have or live with very young children. Third, Table 4 also shows that specifically, it is Surinamese women aged 60 and above that have a larger propensity to be overweight when compared to their male counterparts. Finally, we see that in addition to senior women and seniors who have a chronic condition, those who live in the capital and it’s environs are less likely to be overweight. The predicted probability of being overweight decreases by 7.11% for older individuals who live in Paramaribo.

**TABLE 3:** Factors associated with being overweight/obese – Males and Females, Suriname, 2016–2017.

	Probit-males	Probit - margins	OProbit-males	Probit-females	Probit - margins	OProbit-females
Married/living together	0.238^∗∗∗^	0.0909^∗∗∗^	0.250^∗∗∗^	0.257^∗∗∗^	0.0934^∗∗∗^	0.262^∗∗∗^
	(0.0694)	(0.0262)	(0.0648)	(0.0663)	(0.0238)	(0.0632)
Age	0.0441^∗∗∗^	0.0168^∗∗∗^	0.0484^∗∗∗^	0.0512^∗∗∗^	0.0186^∗∗∗^	0.0536^∗∗∗^
	(0.0122)	(0.0046)	(0.0115)	(0.0111)	(0.0040)	(0.0108)
Age squared	-0.000454^∗∗∗^	-0.000173^∗∗∗^	-0.000476^∗∗∗^	-0.000523^∗∗∗^	-0.000190^∗∗∗^	-0.000547^∗∗∗^
	(0.0001)	(0.0000)	(0.0001)	(0.0001)	(0.0000)	(0.0001)
Lives in capital	-0.0233	-0.00891	-0.0606	0.00105	0.000381	-0.0239
	(0.0672)	(0.0257)	(0.0647)	(0.0669)	(0.0243)	(0.0651)
Lives in interior	0.296	0.113	0.310^∗^	-0.0646	-0.0235	-0.0804
	(0.1910)	(0.0725)	(0.1750)	(0.1650)	(0.0598)	(0.1760)
Asian	-0.649^∗∗^	-0.248^∗∗^	-0.521	-0.0575	-0.0209	-0.0192
	(0.3200)	(0.1220)	(0.3650)	(0.2920)	(0.1060)	(0.2990)
African	-0.571^∗^	-0.218^∗^	-0.395	0.415	0.151	0.447
	(0.3220)	(0.1230)	(0.3660)	(0.2960)	(0.1070)	(0.3010)
Indigenous	-0.286	-0.109	-0.232	0.183	0.0665	0.133
	(0.3870)	(0.1470)	(0.4300)	(0.3410)	(0.1240)	(0.3480)
Mixed	-0.576^∗^	-0.220^∗^	-0.406	0.237	0.0862	0.262
	(0.3250)	(0.1240)	(0.3690)	(0.3000)	(0.1090)	(0.3050)
White	-1.200^∗∗^	-0.458^∗∗^	-0.925^∗^	0.286	0.104	0.42
	(0.5550)	(0.2110)	(0.4950)	(1.1500)	(0.4180)	(1.0350)
Employed	0.114	0.0436	0.17	-0.116	-0.042	-0.0891
	(0.1080)	(0.0411)	(0.1040)	(0.1030)	(0.0375)	(0.0982)
Log of total income	0.00307	0.00117	0.00299	0.00860^∗^	0.00312^∗^	0.00866^∗^
	(0.0037)	(0.0014)	(0.0036)	(0.0047)	(0.0017)	(0.0045)
Active individual	-0.108^∗^	-0.0412^∗^	-0.081	-0.127^∗^	-0.0461^∗^	-0.101
	(0.0629)	(0.0239)	(0.0601)	(0.0665)	(0.0241)	(0.0656)
No of tv sets in household	0.0374	0.0143	0.053	-0.0138	-0.005	0.025
	(0.0447)	(0.0170)	(0.0425)	(0.0458)	(0.0166)	(0.0442)
Level of education	0.0337	0.0129	0.037	-0.0206	-0.00749	-0.0323
	(0.0327)	(0.0124)	(0.0304)	(0.0303)	(0.0110)	(0.0299)
No of 0–5years olds in household	0.0557	0.0213	0.0698	0.0492	0.0179	0.0332
	(0.0556)	(0.0212)	(0.0502)	(0.0482)	(0.0175)	(0.0495)
Household owns agric land	-0.17	-0.0649	-0.116	-0.149	-0.0543	-0.105
	(0.1600)	(0.0610)	(0.1380)	(0.1850)	(0.0672)	(0.1690)
Receives gvt assistance	-0.19	-0.0725	-0.201	-0.18	-0.0654	-0.231^∗∗^
	(0.1470)	(0.0562)	(0.1370)	(0.1140)	(0.0414)	(0.1100)
Has a chronic condition	0.188^∗∗∗^	0.0718^∗∗∗^	0.176^∗∗^	0.438^∗∗∗^	0.159^∗∗∗^	0.440^∗∗∗^
	(0.0726)	(0.0276)	(0.0690)	(0.0711)	(0.0251)	(0.0684)
*N*	1869	1869	1869	1934	1934	1934

Standard errors in parentheses.

*p < 0.10, **p < 0.05, ***p < 0.01.

**TABLE 4:** Factors associated with being overweight/obese - The Young and Old, Suriname, 2016–2017.

	Probit-young	Probit - margins	OProbit-young	Probit-old	Probit - margins	OProbit-old
Female	0.0748	0.0278	0.0629	0.345^∗∗∗^	0.129^∗∗∗^	0.283^∗∗∗^
	(0.0514)	(0.0190)	(0.0486)	(0.0982)	(0.0358)	(0.0921)
Married/living together	0.265^∗∗∗^	0.0985^∗∗∗^	0.281^∗∗∗^	0.0882	0.0329	0.102
	(0.0537)	(0.0196)	(0.0519)	(0.1080)	(0.0402)	(0.1030)
Age	0.0968^∗∗∗^	0.0359^∗∗∗^	0.0907^∗∗∗^	0.0606	0.0226	0.133
	(0.0174)	(0.0064)	(0.0162)	(0.1120)	(0.0418)	(0.1080)
Age squared	-0.00113^∗∗∗^	-0.000419^∗∗∗^	-0.00104^∗∗∗^	-0.0006	-0.00021	-0.00108
	(0.0002)	(0.0001)	(0.0002)	(0.0008)	(0.0003)	(0.0008)
Lives in capital	0.036	0.0134	0.00886	-0.191^∗^	-0.0711^∗^	-0.209^∗∗^
	(0.0560)	(0.0208)	(0.0550)	(0.1010)	(0.0375)	(0.0974)
Lives in interior	0.103	0.0384	0.0687	-0.0019	-0.00069	0.11
	(0.1440)	(0.0535)	(0.1550)	(0.2460)	(0.0917)	(0.2260)
Asian	-0.415^∗^	-0.154^∗^	-0.459^∗∗^	-0.468	-0.174	-0.0865
	(0.2380)	(0.0883)	(0.2210)	(0.3920)	(0.1460)	(0.5020)
African	-0.182	-0.0676	-0.175	-0.0634	-0.0236	0.24
	(0.2410)	(0.0893)	(0.2240)	(0.4000)	(0.1490)	(0.5110)
Indigenous	-0.0838	-0.0311	-0.228	-0.313	-0.117	0.0458
	(0.2930)	(0.1090)	(0.2860)	(0.4770)	(0.1780)	(0.5570)
Mixed	-0.296	-0.11	-0.327	-0.033	-0.0123	0.366
	(0.2440)	(0.0907)	(0.2270)	(0.4060)	(0.1510)	(0.5140)
White	-1.265^∗∗^	-0.469^∗∗^	-0.986^∗∗∗^	0	0	4.833^∗∗∗^
	(0.5500)	(0.2030)	(0.3570)	(.)	(.)	(0.5750)
Employed	0.0175	0.0065	0.0285	0.00494	0.00184	0.118
	(0.0822)	(0.0305)	(0.0788)	(0.1650)	(0.0613)	(0.1520)
Log of total income	0.00502	0.00186	0.00587^∗^	-0.0003	-0.00011	-0.00379
	(0.0032)	(0.0012)	(0.0031)	(0.0082)	(0.0031)	(0.0079)
Active individual	-0.120^∗∗^	-0.0444^∗∗^	-0.0936^∗^	-0.0014	-0.00053	0.00874
	(0.0511)	(0.0189)	(0.0509)	(0.1010)	(0.0377)	(0.0967)
No of tv sets in household	0.00392	0.00145	0.0412	0.07	0.0261	0.066
	(0.0379)	(0.0141)	(0.0369)	(0.0663)	(0.0247)	(0.0609)
Level of education	-0.00808	-0.003	-0.0158	0.0244	0.0091	0.0303
	(0.0258)	(0.0096)	(0.0250)	(0.0462)	(0.0172)	(0.0449)
No of 0–5years olds in household	0.0839^∗∗^	0.0311^∗∗^	0.0732^∗^	-0.171	-0.0637	-0.128
	(0.0404)	(0.0150)	(0.0386)	(0.1090)	(0.0403)	(0.1050)
Household owns agric land	-0.156	-0.0578	-0.102	-0.268	-0.1	-0.207
	(0.1290)	(0.0480)	(0.1130)	(0.4280)	(0.1600)	(0.3500)
Receives gvt assistance	-0.134	-0.0498	-0.189^∗∗^	-0.347	-0.129	-0.25
	(0.0966)	(0.0358)	(0.0932)	(0.3040)	(0.1130)	(0.2380)
Has a chronic condition	0.366^∗∗∗^	0.136^∗∗∗^	0.366^∗∗∗^	0.311^∗∗∗^	0.116^∗∗∗^	0.291^∗∗∗^
	(0.0601)	(0.0219)	(0.0573)	(0.0961)	(0.0352)	(0.0898)
*N*	3,025	3,025	3,025	776	776	778

Standard errors in parentheses

*p < 0.10, **p < 0.05, ***p < 0.01.

Finally, as a robustness check, we also create a dummy variable indicating whether an individual is obese using previous ethnic and sex-specific cut-oﬀ values and re-run our probit regression using this variable as the dependent variable [20]. The results from this estimation cannot be directly compared to our previous probit models in Tables 2–4 because the dependent variable used for these models is a binary variable indicating whether an individual is either overweight. To make our results comparable to the estimates using previous cut-oﬀ values [20], we construct another binary variable indicating whether an individual is obese or not using the standard WHO adult obesity cut-oﬀ value which is 30.0. Apart from the estimates for Female, Asian, African, Indigenous and Chronic which change appreciably because the optimal cut-oﬀ values take these factors directly into account, the qualitative conclusions from our study largely remain the same [20].

## Discussion

Our findings show that women, especially senior women and women who earn relatively higher incomes, are more at risk than their male counterparts to be overweight. Using a quantile regression analysis, some find this to be true for Turkish women especially at the highest quantiles of BMI [24]. Our results also indicate that the marital status of an individual can be strongly associated with his or her BMI. In particular, we find that, especially among the younger adult population, individuals that are married or living together are more likely to be obese. This result is understandable since persons who are living together are likely happier and have more income available to enjoy certain luxuries that single person cannot access easily.

We also find that the propensity to be overweight increases until age 48 after which the relationship becomes negative. This is not surprising as general health and physical activity levels decrease with age. The fact that having a chronic illness such as diabetes and high blood pressure is strongly associated with being overweight is not surprising. Several researchers suggest that these diseases are either caused by obesity or may result in becoming overweight [27–29].

The result that active individuals are generally less likely to be overweight is corroborated by the results of others who find that each hour of moderate physical activity is associated with a 0*.*085 kg/m^2^ decrease in BMI among adults in rural South India [30]. Using data from 15 member states of the European Union, one study also finds that obesity among adults is strongly associated with a sedentary lifestyle and physical inactivity during their leisure time [31]. These findings are consistent with the view that even a modest level of physical activity may generate health benefits. This is especially true today since modernization and technology have contributed to a general decline in physical activity [32–34].

Interestingly, our findings indicate that individuals who benefit from government social safety net programs are less likely to be associated with being overweight or obese. This may be a result of the fact that these individuals are usually the most vulnerable in the society and hence can barely aﬀord to meet their basic needs. The fact that beneficiaries of public safety net programs are less likely to be overweight is really an interesting outcome for the literature. Actually, similar to other authors [24, 34], we find some evidence that people with higher income levels tend to have higher BMI levels although unlike these papers, this result is not highly significant in our case.

The results of a qualitative study conducted on Afro-Caribbean women corroborates our result that being overweight is likely to increase for young adults who are parents [35]. The study finds that being active tends to compete with family and caring responsibilities. Hence, adults with young children tend to be less active than adults who do not have any young children and therefore have a higher propensity of being overweight. This is likely to be truer for women than for men as other previous studies confirm that the responsibility of childcare falls predominantly on women in Caribbean countries [36–38].

In concluding, the high prevalence of obesity and overweight in the Caribbean could cause serious public health issue if not addressed appropriately. In this study for instance, using Surinamese data, we find that almost 60% of our sample is overweight. Of course, it is possible that the overweight situation can be explained by nutritional transition given that such guidelines currently exist in Suriname [39]. It is therefore prudent for policymakers to develop a national strategy to help health care systems cope with the outcomes this phenomenon and to tackle the risk factors that have the greatest impacts on individual BMI.

Due to data limitations, the analysis did include factors such as dietary patterns, smoking and drinking habits, sleep patterns, which are strongly associated with being overweight. Further, residual confounding and reverse causation may exist due to the cross-sectional design of the study. Nevertheless, the results highlight the need to design and implement pointed policies aimed at reducing obesity. However, the success of such policies is likely to hinge on taking account of the factors that are associated with being overweight. The results of this study can also be used to adjust current nutritional guidelines. Further, given the noted obesity prevalence in other Caribbean countries, these results can influence action in these countries where obesity policies do not exist.
